# Combined impact of paced QRS duration and right ventricular pacing burden on heart failure admission: Identifying clinical progression to stage C/D in pacemaker recipients

**DOI:** 10.1016/j.hroo.2026.07.013

**Published:** 2026-07-21

**Authors:** Takeshi Ueyama, Shoya Sugita, Masaaki Yoshida, Tomoyuki Uchida, Yufuko Nishida, Chihiro Yamada, Fumiaki Nakao, Yasuhiro Ikeda

**Affiliations:** 1 Department of Cardiology, Yamaguchi Prefectural Grand Medical Center, Hofu, Japan; 2 Department of Clinical Engineering, Yamaguchi Prefectural Grand Medical Center, Hofu, Japan

**Keywords:** Paced QRS duration, Right ventricular pacing burden, Heart failure admission, Pacing-induced cardiomyopathy, Remote monitoring

## Abstract

**Background:**

Right ventricular (RV) pacing-induced dyssynchrony can lead to progressive left ventricular (LV) dysfunction. Although a decline in LV ejection fraction (LVEF) after pacemaker implantation is common, the transition to symptomatic heart failure (HF) requiring hospitalization (HF admission [HFA]) represents a more clinically meaningful progression to stages C/D.

**Objective:**

To determine whether paced QRS (pQRS) duration and RV pacing (RVp) burden independently or jointly predict HFA in pacemaker recipients followed with remote monitoring.

**Methods:**

We retrospectively analyzed 200 pacemaker recipients actively followed with remote monitoring. pQRS at implantation and RVp during follow-up were evaluated as markers of baseline electrical dyssynchrony and cumulative pacing exposure. The primary endpoint was HFA, whereas the secondary endpoint was all-cause mortality.

**Results:**

During a median follow-up of 4.5 years, 27 patients (13.5%) developed HFA. Patients with HFA had longer pQRS (171 ± 18 vs 156 ± 20 ms, *P* < .001) and higher RVp (median 99% vs 38%, *P* = .002). In a multivariable Cox model, both pQRS of ≥168 ms (hazard ratio, 2.98, *P* = .011) and RVp of ≥20% (hazard ratio, 7.16, *P* = .009) and age independently predicted HFA. Neither pQRS nor RVp predicted all-cause mortality, which was associated only with age.

**Conclusion:**

pQRS and RVp jointly identify patients at high risk of progression from clinical to symptomatic HF (stages C/D). Although mortality in this population is primarily age-driven, HFA reflects a modifiable consequence of pacing-related dyssynchrony. Combined assessment of pQRS and RVp may facilitate early identification of high-risk patients who could benefit from closer monitoring or alternative pacing strategies.

**Key Findings**

▪ Prolonged paced QRS (pQRS) duration (≥168 ms) and high right ventricular pacing (RVp) burden (≥20%) independently predict heart failure (HF) admission in pacemaker recipients.
▪ The combination of prolonged pQRS and high RVp identifies a high-risk subgroup with early progression to symptomatic HF (stage C/D).
▪ Pacing-related dyssynchrony strongly influences morbidity (HF hospitalization) but does not significantly affect all-cause mortality.
▪ RVp burden shows a dose–response relationship with HF risk, with higher thresholds (≥40% and ≥60%) also associated with increased events.
▪ Combined assessment of pQRS and RVp may guide early intervention, device programming, and pacing strategy optimization in the remote monitoring era.

## Introduction

Right ventricular pacing (RVp) is the standard therapy for symptomatic bradyarrhythmias; however, it can induce electrical and mechanical dyssynchrony.^1^ This dyssynchrony often leads to progressive decline in left ventricular ejection fraction (LVEF), a condition known as pacing-induced cardiomyopathy (PICM).^1^, ^2^, ^3^ Although a post-implantation decline in LVEF is frequently observed, the most clinically significant transition is the development of symptomatic heart failure (HF) requiring hospitalization (HFA). In the clinical course of HF, this represents a critical progression from stage B (asymptomatic phase) to stage C or D (symptomatic HF) in the American College of Cardiology/American Heart Association classification.^4^

Therefore, the early identification of patients at high risk for clinical HF progression—rather than merely detecting a decline in LVEF—is of paramount importance. However, reliable predictors of HFA in real-world pacemaker recipients remain insufficiently understood.

Previous studies have examined individual pacing-related factors. A prolonged paced QRS (pQRS), duration a marker of electrical dyssynchrony, has been associated with HF events and PICM.^5^, ^6^, ^7^ Separately, a high RV pacing (RVp) burden, representing cumulative exposure to dyssynchrony, has been linked to PICM and LV dysfunction.^8^, ^9^, ^10^ Yet these factors have largely been evaluated in isolation. Importantly, few studies have assessed clinical HF admission as a primary outcome, and fewer still have integrated both pQRS (as baseline electrical dyssynchrony) and RVp (as cumulative exposure) to evaluate their synergistic impact on HF progression.^11^^,^^12^ Furthermore, the relationship between pacing-related dyssynchrony and mortality remains poorly defined.

In the current era of remote monitoring (RM) and diverse pacing modalities—including leadless pacemakers—there is a growing need for simple, widely available markers that can identify patients at risk for clinically significant HF. Therefore, the present study aimed to determine whether the combination of a prolonged pQRS at implantation and a high RVp during follow-up predicts HFA in pacemaker recipients. We further evaluated whether these pacing-related parameters are associated with all-cause mortality, to clarify whether dyssynchrony primarily affects morbidity or mortality in this population.

## Methods

### Study design and population

This was a single-center retrospective cohort study conducted at Yamaguchi Prefectural Grand Medical Center. We identified 550 patients who were actively followed with an RM system at our institution between October 2008 and June 2025. Among them, 200 consecutive patients without pre-existing HF and with sufficient follow-up data were included in the final analysis (Figure 1). The median follow-up duration for the entire cohort was 1647 days (4.5 years). Patients who were followed at other hospitals or who declined RM were not included. Some patients had both sinus node dysfunction and atrioventricular (AV) block. For baseline classification, the primary clinical indication judged by the implanting physician was used, whereas the presence of AV block was separately reported in Table 1. The retrospective, single-center study was conducted in accordance with the Declaration of Helsinki and current pacing guidelines.^13^, ^14^, ^15^ Written informed consent was waived due to the retrospective nature of the study. The research reported in this paper adhered to the ethical guidelines of the Declaration of Helsinki and its later amendments. The study protocol was approved by the institutional ethics committee of Yamaguchi Prefectural Grand Medical Center (approval number: 2025-019), and an opt-out process was implemented by publicly announcing the study on the hospital website.

**Figure 1:**
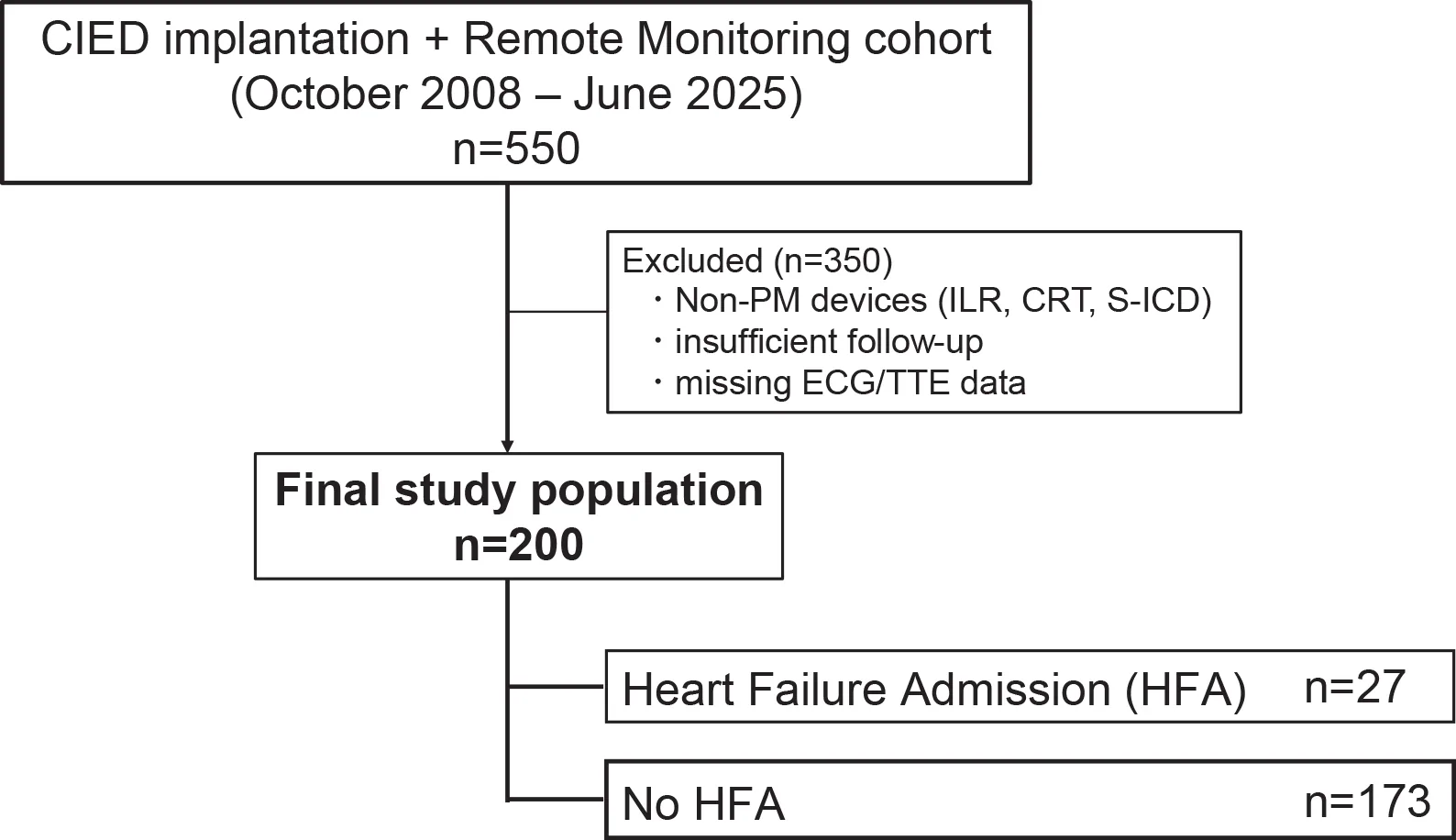
Study flow diagram. Flowchart illustrating patient selection from the initial cohort of 550 individuals with cardiac implantable electronic devices and RM. After excluding patients with non-pacemaker devices, insufficient follow-up, or missing electrocardiogram or echocardiographic data, 200 patients were included in the final analysis. Among them, 27 experienced heart failure admission during follow-up.

**Table 1:** Baseline and follow-up characteristics of the study population

Variable	All (n = 200)	HFA (−) n = 173	HFA (+) n = 27	*P*-value
Demographic and device indication				
Age at implantation, years	74 ± 11	74 ± 11	76 ± 11	.518
Men, n (%)	114 (57%)	97 (56%)	17 (59%)	.538
AV block, n (%)	109 (55%)	93 (54%)	16 (59%)	.680
Pacing and echocardiographic parameters				
Intrinsic QRS (ms)	102 ± 21	101 ± 12	106 ± 27	.220
Paced QRS (ms)	158 ± 20	156 ± 20	171 ± 18	<.001
LVEDD (mm)	48 ± 8	48 ± 8	50 ± 11	.293
LVESD (mm)	33 ± 10	32 ± 9	37 ± 13	.012
Baseline LVEF (%)	60 ± 13	61 ± 13	59 ± 12	.451
Follow-up LVEF (%)	56 ± 13	58 ± 12	46 ± 18	<.001
RVp, % (IQR)	61.6 (0.2–99.9)	38 (0.1–99.8)	99 (86.5–99.9)	.002
Comorbidities				
Atrial fibrillation, n (%)	75 (38%)	61 (35%)	14 (52%)	.314
Chronic kidney disease, n (%)	32 (16%)	26 (15%)	6 (22%)	.396
Hemodialysis, n (%)	8 (4%)	6 (3%)	2 (7%)	.295
Cardiomyopathy, n (%)	21 (11%)	17 (10%)	4 (15%)	.496
COPD, n (%)	6 (3%)	4 (2%)	2 (7%)	.187
Diabetes mellitus, n (%)	61 (31%)	51 (29%)	10 (37%)	.501
Hypertension, n (%)	108 (54%)	93 (54%)	15 (56%)	1.000
Sleep apnea, n (%)	15 (8%)	12 (7%)	3 (11%)	.433
CPAP, n (%)	8 (4%)	5 (3%)	3 (11%)	.078
Valvular heart disease, n (%)	28 (14%)	20 (12%)	8 (30%)	.031
Prior cardiac surgery, n (%)	22 (11%)	15 (9%)	7 (26%)	.016
Prior PCI, n (%)	34 (17%)	28 (16%)	6 (22%)	.417
Prior RFCA, n (%)	30 (15%)	25 (14%)	6 (22%)	.254

Values are mean ± standard deviation, median (interquartile range), or n (%). Follow-up LVEF represents the most recent echocardiographic assessment during follow-up.

AV = atrioventricular; CPAP = continuous positive airway pressure; COPD = chronic obstructive pulmonary disease; HFA = heart failure association; IQR = interquartile range; LVEDD = left ventricular end-diastolic diameter; LVEF = left ventricular ejection fraction; LVESD = left ventricular end-systolic diameter; PCI = percutaneous coronary intervention; RFCA = radiofrequency catheter ablation; RVp = right ventricular pacing.

### Data collection and pacing parameters

Baseline clinical characteristics, including age, sex, and the indication for pacing, were obtained from electronic medical records. pQRS was measured from a standard 12-lead electrocardiogram recorded immediately or shortly after device implantation. RVp sites included the RV septum (most common), RV apex, with a minority of patients receiving His-bundle pacing (HBP), left bundle branch area pacing (LBBAP), or epicardial leads. Devices were programmed to prioritize intrinsic AV conduction and minimize unnecessary ventricular pacing. AV delay was adjusted when clinically indicated, occasionally with echocardiographic guidance, although routine AV delay optimization was not performed in all patients. The RVp was defined as the percentage of ventricular pacing during the follow-up period, as recorded using the device’s daily RM data. Consistent with previous literature and our receiver operating characteristic (ROC) analysis, we defined a prolonged pQRS as ≥168 ms and a high RVp as ≥20%.^8^ RVp was analyzed both as a continuous variable and as a categorical variable.

### Echocardiographic measurements and evaluation

Transthoracic echocardiography was performed at baseline (pre-implantation) and during the follow-up period. LVEF was measured using the biplane Method of Discs (Simpson’s rule). A progressive decline in LVEF was defined as an absolute decrease of ≥10% from the baseline value. LV end-diastolic diameter (LVEDD) and end-systolic diameter (LVESD) were measured at baseline and during follow-up when available. RV dimension (RVd) was not routinely measured in our clinical practice and was available only in a small subset of patients. Follow-up LVEF represents the most recent echocardiographic assessment during the study period.

### Study endpoints

The primary endpoint was HFA, defined as an unplanned hospitalization requiring intravenous diuretic therapy for worsening HF symptoms (corresponding to HF stage C/D).^4^ The secondary endpoint was all-cause mortality. Endpoint events were identified through electronic medical records, RM alerts, and regular follow-up visits.

#### Risk group classification

To evaluate the combined impact of baseline electrical dyssynchrony and cumulative pacing exposure, patients were categorized into 3 risk groups: high-risk: both pQRS of ≥168 ms and RVp of ≥20%; intermediate-risk: either pQRS of ≥168 ms or RVp of ≥20%; and low-risk: neither factor present.

### Statistical analysis

Continuous variables are expressed as mean ± standard deviation or median with interquartile range (IQR) and were compared using the Student’s *t*-test or Mann–Whitney U test, as appropriate. Categorical variables were compared using the χ^2^ test or Fisher’s exact test. Kaplan–Meier curves were constructed to estimate the cumulative incidence of HFA and all-cause mortality, with differences assessed using the log-rank test. Multivariable Cox proportional hazards models were used to identify independent predictors of endpoints. For the multivariable Cox model, we included baseline clinical characteristics and the percentage of RVp during the follow-up period. Follow-up–dependent echocardiographic parameters (such as post-implant LVEF or LVEF decline) were excluded because they act as longitudinal mediators rather than baseline predictors, whereas RVp was incorporated as a measure of cumulative exposure established before the primary endpoint. The final model included pQRS of ≥168 ms, RVp of ≥20%, age, sex, and baseline LVEF. A 2-sided *P*-value of <.05 was considered statistically significant. All statistical analyses were performed using EZR Version 1.66 (Saitama Medical Center, Jichi Medical University, Saitama, Japan), which is a graphical user Interface for R (The R Foundation for Statistical Computing, Vienna, Austria).

## Results

### Baseline characteristics

The study included 200 patients (mean age, 74 ± 11 years; 57% male). The primary indications for pacemaker implantation were sick sinus syndrome (42%) and AV block (48%). Some patients had both sinus node dysfunction and AV block. In such cases, the primary clinical indication judged by the implanting physician was used for classification in the text, whereas Table 1 reports the presence of AV block irrespective of the primary indication. Baseline clinical characteristics and group comparisons are summarized in Table 1. Baseline LVEF was 60% ± 13%, with no significant difference between patients with and without HFA. Baseline LVEDD and LVESD were similar between groups (LVEDD: 48 ± 8 mm vs 50 ± 11 mm, *P* = .293; LVESD: 32 ± 9 mm vs 37 ± 13 mm, *P* = .012). The median RVp in the overall cohort was 61.6% (IQR, 0.2–99.9). Patients who developed HFA had a lower follow-up LVEF (46% ± 18% vs 58 ± 12%, *P* < .001), longer pQRS duration, and a higher RVp compared with those without HFA. Valvular heart disease and prior cardiac surgery were more frequent in the HFA group (30% vs 12%, *P* = .031; 26% vs 9%, *P* = .016, respectively). Valvular heart disease and prior cardiac surgery were more frequent in the HFA group (30% vs 12%, *P* = .031; 26% vs 9%, *P* = .016, respectively). The median follow-up duration for the entire cohort was 1647 days (4.5 years). Most patients were paced at the RV septum (HFA–: 101, HFA+: 5) or the RV apex (HFA–: 63, HFA+: 19). A small number of patients received conduction system pacing, including HBP (HFA–: 2, HFA+: 0) and LBBAP (HFA–: 4, HFA+: 2). Only 2 patients with LBBAP experienced HFA, and none of the patients with HBP were hospitalized for HF.

### Follow-up echocardiography

In the overall cohort, LVEF modestly decreased from baseline to follow-up (60% ± 13% to 56% ± 13%, *P* < .001). LVEDD showed a small but statistically significant reduction (48.3 ± 8.2 mm to 47.2 ± 9.1 mm, *P* = .013), whereas LVESD remained unchanged (33.0 ± 9.6 mm to 32.9 ± 9.6 mm, *P* = .827). RVd was available only in a limited number of patients (n = 6–12) and was therefore not included in statistical comparisons. These detailed echocardiographic changes are summarized in Supplemental Table 1.

### Primary endpoint: HF admission

During the follow-up period, 27 patients (13.5%) reached the primary endpoint of HFA. The median time from pacemaker implantation to the first HFA was 1241 days (3.4 years). Patients who experienced HFA had significantly longer pQRS duration compared with those who did not (171 ± 18 vs 156 ± 20 ms; *P* < .001). Furthermore, the HFA group exhibited a substantially higher RVp (median 99% [IQR, 74%–100%] vs 38% [IQR, 5%–95%]; *P* = .002). Regarding echocardiographic changes, the HFA group showed lower post-implant LVEF and a higher incidence of LVEF decline of ≥10% compared with the non-HFA group (Figure 2).

**Figure 2:**
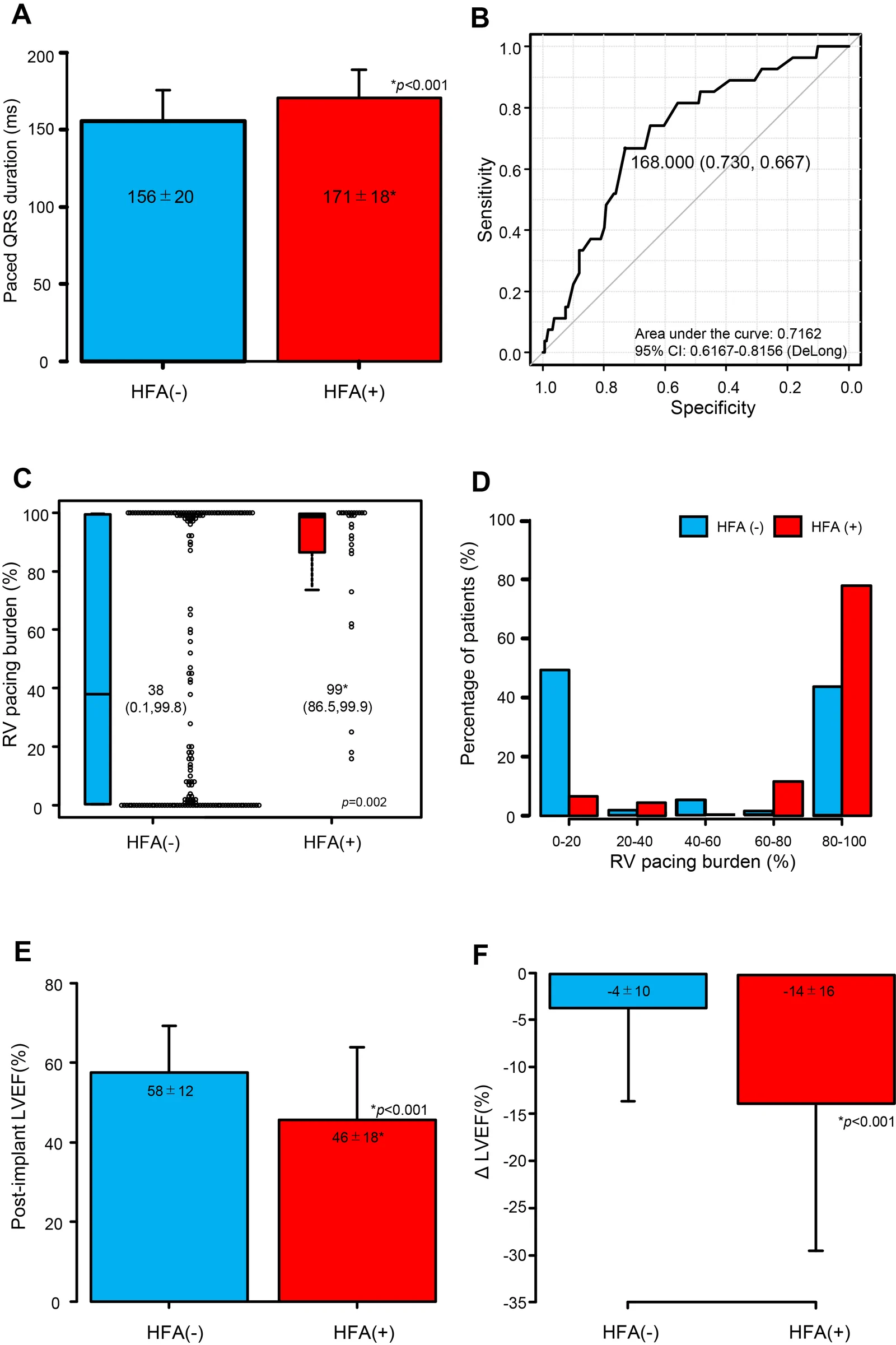
Distribution of pacing-related and echocardiographic parameters in patients with and without heart failure admission (HFA). **A:** Comparison of paced QRS duration (pQRS) in patients with and without HFA. **B:** Receiver operating characteristic curve for pQRS predicting HFA (area under the curve 0.716). **C:** Distribution of right ventricular pacing burden (RVp) in patients with and without HFA, shown using a dot plot with an adjacent boxplot to illustrate individual data points and the skewed distribution. **D:** Distribution of RVp categories (0%–20%, 20%–40%, 40%–60%, 60%–80%, and 80%–100%) in patients with and without HFA, shown as percentages. **E:** Post-implant left ventricular ejection fraction (LVEF) in patients with and without HFA. **F:** Change in LVEF (ΔLVEF) after pacemaker implantation in patients with and without HFA, shown as mean ± standard deviation.

### Predictors of HFA

To evaluate the robustness of our findings, we performed sensitivity analyses using alternative RVp thresholds other than the primary cutoff of ≥20%. RVp of ≥40% (hazard ratio [HR], 4.62; 95% confidence interval [CI], 1.36–15.66) and RVp of ≥60% (HR, 5.12; 95% CI, 1.51–17.42) remained significantly associated with HFA. In the multivariable Cox model including baseline variables (pQRS ≥168 ms, RVp ≥ 20%, age, sex, and baseline LVEF), both prolonged pQRS (HR, 2.98; 95% CI, 1.28–6.94, *P* = .011) and high RVp (HR, 7.16; 95% CI, 1.63–31.44, *P* = .009) remained independent predictors of HFA. Age was also associated with increased risk (HR, 1.04 per year, *P* = .035), whereas sex and baseline LVEF were not significant (Table 2).

**Table 2:** Multivariable Cox regression analysis for HFA

Variable	HR	95% CI	*P*-value
pQRS ≥168 ms	2.98	1.28–6.94	.011
RVp ≥20%	7.16	1.63–31.44	.009
Age (per 1 year)	1.04	1.00–1.09	.035
Male sex	1.00	0.42–2.39	.994
Baseline LVEF (%)	0.97	0.94–1.00	.056

Concordance = 0.794.

CI = confidence interval; HR = hazard ratio; LVEF = left ventricular ejection fraction; pQRS = paced QRS duration; RVp = right ventricular pacing.

Multivariable Cox model includes baseline variables only.

### Combined impact of pQRS and RVp on HFA

Kaplan–Meier analysis demonstrated a clear separation in HFA-free survival among the 3 risk groups (log-rank *P* < .001; Figure 3). The high-risk group (both pQRS ≥168 ms and RVp ≥20%) showed the highest and earliest incidence of HFA, followed by the intermediate-risk group. This result indicates a synergistic effect between baseline electrical dyssynchrony (pQRS) and cumulative pacing exposure (RVp) on the progression to symptomatic HF (Stage C/D).

**Figure 3:**
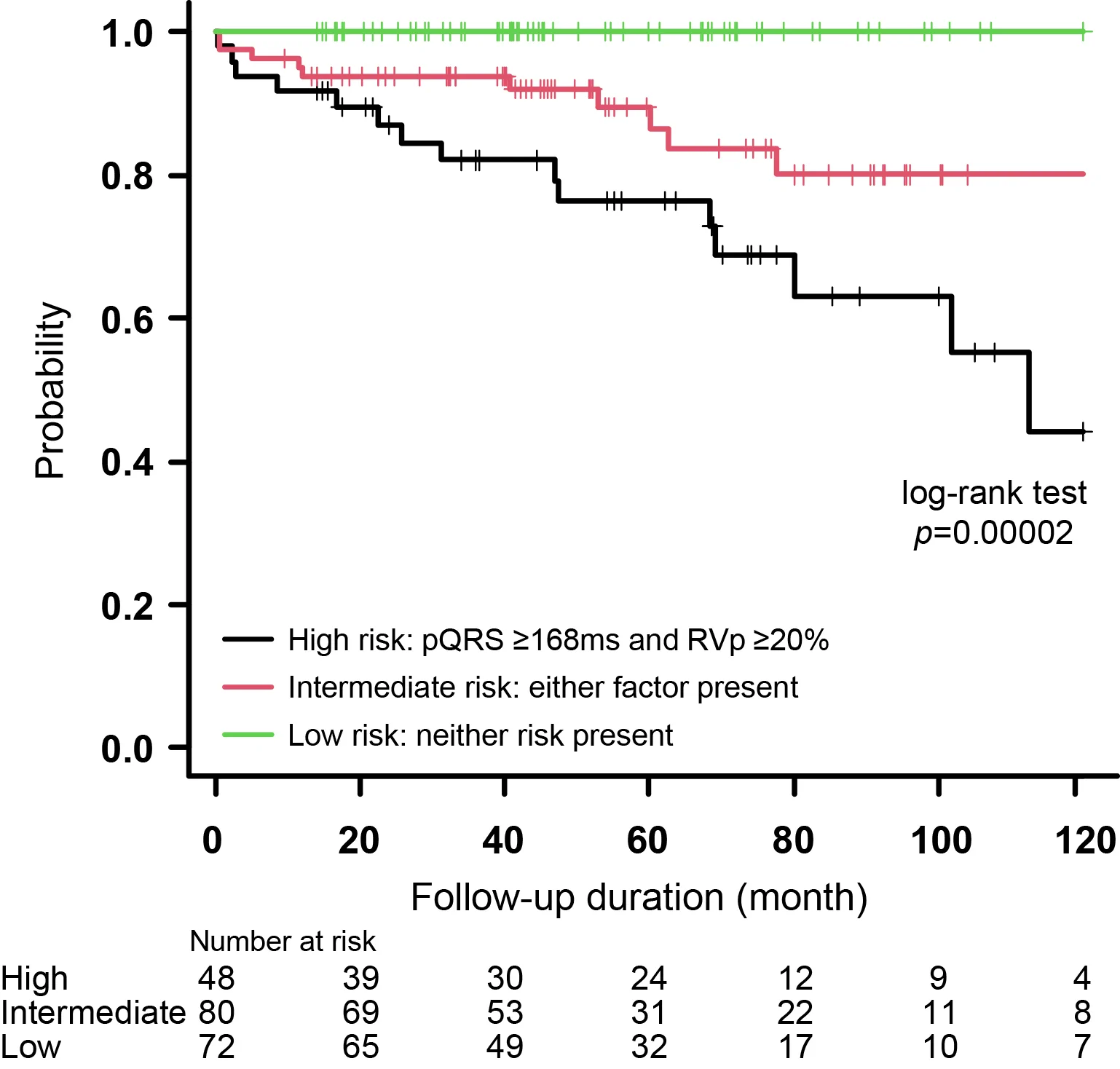
Kaplan–Meier curves for heart failure admission (HFA) according to combined paced QRS duration (pQRS) and right ventricular pacing (RVp) risk groups. Patients were stratified into 3 groups based on pQRS duration (≥168 ms) and RVp burden (≥20%): High-risk: both factors present; intermediate-risk: either factor present; low-risk: neither factor present. The high-risk group showed the earliest and highest incidence of HFA, demonstrating a synergistic effect of baseline electrical dyssynchrony and cumulative pacing exposure (log-rank *P* < .001).

### Secondary endpoint: All-cause mortality

During the follow-up, 32 patients (16.0%) died. Unlike the results for HFA, the 3 risk groups showed no significant difference in all-cause mortality (log-rank *P* = .115; Figure 4). In the multivariable Cox model for all-cause mortality using the same baseline variables as the HFA analysis, age was the only independent predictor of death (HR, 1.10 per year; 95% CI, 1.04–1.17, *P* = .00078). In contrast, neither pQRS of ≥168 ms nor RVp of ≥20% was associated with mortality, and sex and baseline LVEF were also non-significant (Table 3).

**Figure 4:**
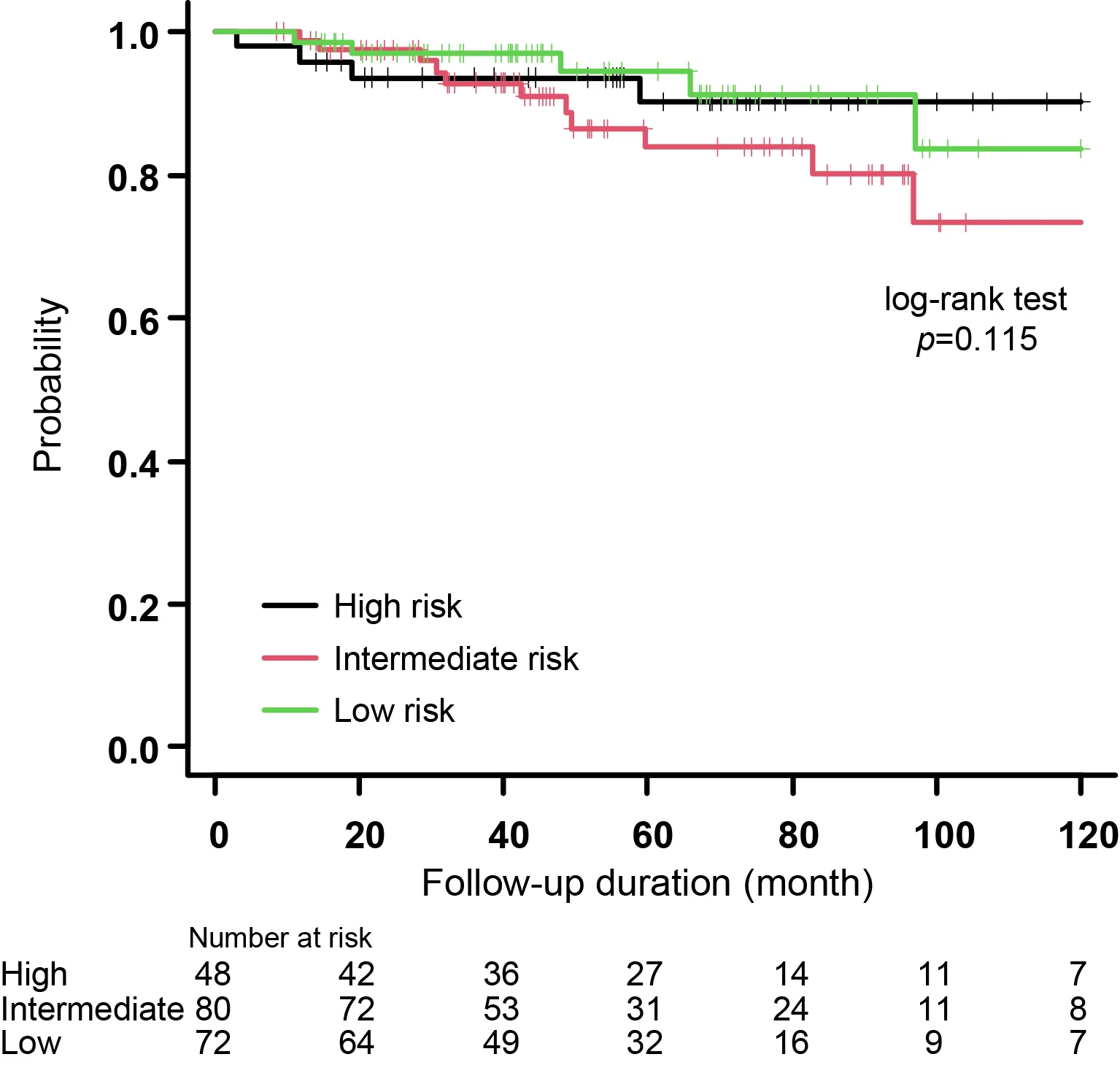
Kaplan–Meier curves for all-cause mortality according to combined paced QRS duration and right ventricular pacing risk groups. Despite clear differences in heart failure admission, the 3 risk groups showed no significant difference in all-cause mortality. Mortality curves overlapped substantially, indicating that pacing-related dyssynchrony influenced morbidity but not mortality in this cohort (log-rank *P* = .115).

**Table 3:** Multivariable Cox regression analysis for all-cause mortality

Variable	HR	95% CI	*P*-value
pQRS ≥168 ms	1.20	0.48–2.98	.696
RVp ≥20%	0.95	0.39–2.27	.901
Age (per 1 year)	1.10	1.04–1.17	.00078
Male sex	0.55	0.22–1.40	.209
Baseline LVEF (%)	1.00	0.96–1.04	.952

Concordance = 0.752.

CI = confidence interval; HR = hazard ratio; LVEF = left ventricular ejection fraction; pQRS = paced QRS duration; RVp = right ventricular pacing. The same baseline variables used in the HFA model were applied for mortality analysis.

## Discussion

### Major findings

The primary finding of this study is that the combination of a prolonged pQRS duration (≥168 ms) at implantation and a high RVp (≥20%) during follow-up is a potent predictor of HFA in pacemaker recipients. Although all-cause mortality was primarily driven by age, HFA represented a modifiable consequence of pacing-induced dyssynchrony. These results suggest that identifying electrical dyssynchrony early and monitoring pacing exposure can capture the clinical progression from stage B to stage C/D HF.

In the overall cohort, LVEDD showed a small but significant reduction after pacemaker implantation, suggesting that many patients benefited from the hemodynamic effects of pacing, including correction of bradycardia and improved loading conditions. In contrast, patients who developed HFA exhibited larger LVESD at baseline and a marked decline in LVEF during follow-up, indicating that pacing-induced dyssynchrony outweighed these beneficial effects in this subgroup. These findings highlight that conventional RVp may provide modest reverse remodeling in many patients, whereas a subset is vulnerable to adverse remodeling leading to HFA. These echocardiographic findings support the notion that pacing may confer hemodynamic benefit in many patients, whereas those who develop HFA represent a subgroup in whom pacing-induced dyssynchrony predominates over these beneficial effects. Detailed echocardiographic changes are summarized in Supplemental Table 1.

### Electrical dyssynchrony and pacing exposure

Although 42% of our cohort had sinus node dysfunction, a substantial proportion exhibited progressive or intermittent AV conduction disease during follow-up, leading to a higher-than- expected RVp burden. This phenomenon has been reported in elderly populations, in whom intrinsic conduction often deteriorates over time. Our findings are consistent with prior meta-analytic evidence showing that both pQRS and RVp are key risk factors for PICM.^1^ The synergistic effect of pQRS and RVp observed in our study provides a more comprehensive risk stratification than evaluating either factor alone. A prolonged pQRS duration reflects significant electrical dyssynchrony, which serves as a substrate for mechanical inefficiency.^5^, ^6^, ^7^ High RVp has been linked to LV dysfunction and PICM.^8^, ^9^, ^10^ When this substrate is coupled with frequent RVp—representing chronic exposure to this inefficiency—the risk of symptomatic HF amplifies. This is consistent with previous reports, such as Chen et al,^7^ who showed that pQRS predicts HF events, and Kiehl et al,^8^ who identified RVp of ≥20% as a threshold for PICM. Our study integrates these 2 dimensions, demonstrating that patients with both factors exhibit the steepest decline in HFA-free survival. Sensitivity analyses using alternative RVp thresholds demonstrated a consistent and robust association with HFA. When RVp ≥40% and ≥60% were used instead of 20%, the risk of HFA remained significantly elevated, supporting a dose-response relationship between cumulative RVp exposure and HF progression. Although higher RVp values visually appear to cluster among patients with HFA, our ROC analysis indicated that risk begins to rise well below 60%, with an optimal cutoff approximately 16%. These findings reinforce that even moderate levels of RVp can contribute to symptomatic HF in susceptible patients. Therefore, RVp ≥20% remains a clinically meaningful and literature-supported threshold.

### Morbidity vs mortality

Valvular heart disease and prior cardiac surgery were more common in patients who developed HFA, likely reflecting a greater burden of underlying structural heart disease in this group. However, these factors did not remain independent predictors in the multivariable analysis. In the multivariable analysis, both prolonged pQRS and high RVp were independent predictors of HFA, even after adjusting for age, sex, and baseline LVEF. These findings indicate that pacing-related electrical dyssynchrony and cumulative pacing exposure are key determinants of clinical HF progression, independent of baseline LV systolic function. Pacing-related parameters (pQRS and RVp) were not associated with all-cause mortality, which was driven almost entirely by age. This contrasts with the HFA model, which indicates that pacing-induced dyssynchrony primarily affects morbidity rather than mortality. Prior studies have shown that RVp is associated with HF hospitalization but not necessarily mortality.^11^^,^^12^ In an elderly population with multiple comorbidities, mortality may be determined by broader age-related factors rather than pacing exposure itself. This finding suggests that pacing-related dyssynchrony primarily affects morbidity and quality of life through repeated hospitalizations. In an aging population with multiple comorbidities, reducing HFA through early identification of high-risk patients is a crucial clinical goal, even if it does not directly extend overall survival. Medications that affect AV conduction were not included as predictors because they primarily influence the indication for pacemaker implantation rather than the subsequent risk of HFA. Furthermore, medication regimens frequently changed during follow-up, making them unsuitable as baseline predictors in a Cox model. Importantly, RVp reflects the net effect of intrinsic conduction, device programming, and any medication-related influences, and therefore serves as a more comprehensive and stable exposure variable.

### Clinical implications and the role of RM

In the era of RM, the combined assessment of pQRS and RVp enables early identification of patients at high risk for clinical progression from stage B to stage C/D HF. For these high-risk individuals, device programming aimed at minimizing unnecessary RVp represents an immediate and practical strategy to reduce pacing-induced dyssynchrony. In addition, closer clinical and echocardiographic follow-up, together with optimization of guideline-directed HF therapy, may help prevent the development of symptomatic HF requiring hospitalization. For patients who remain highly paced with evidence of dyssynchrony despite these measures, alternative pacing strategies—such as biventricular cardiac resynchronization therapy or conduction system pacing—should be considered. Although HBP may be limited by higher capture thresholds, LBBAP has emerged as a more stable and physiologic option, supported by recent multicenter data and landmark studies.^16^, ^17^, ^18^, ^19^, ^20^, ^21^, ^22^ These strategies may be particularly relevant in an aging pacemaker population in whom morbidity, rather than mortality, is the dominant consequence of pacing-induced dyssynchrony.

### Limitations

This study has several limitations. First, this was a single-center retrospective study with a relatively small number of HFA events, which may limit the statistical power and generalizability. Second, echocardiographic follow-up intervals varied among patients, although the median interval of 3 years was sufficient to detect meaningful LVEF changes. RVd was not routinely measured and was available only in a small subset of patients, thereby limiting our ability to evaluate RV remodeling. However, LV dimensions and LVEF were available in the majority of patients and are presented in Supplemental Table 1. Third, although we accounted for several baseline variables, unmeasured confounders, such as changes in medication during follow-up, might have influenced the outcomes. Finally, we did not adjudicate the causes of death; thus, the relationship between pacing and cardiovascular mortality remains to be clarified.

## Conclusion

Paced QRS duration and RVp jointly identify patients at high risk for clinical HF progression from stage B to stage C/D. Although mortality in this population is primarily driven by age, HFA reflects a modifiable consequence of pacing-related dyssynchrony. Combined assessment of pQRS and RVp may facilitate early identification of high-risk patients who might benefit from closer monitoring or alternative pacing strategies.

## Declaration of generative AI and AI-assisted technologies in the writing process

The authors used generative AI tools (Microsoft Copilot, Google Gemini, and OpenAI ChatGPT) to assist with language refinement, organization of text, and improvement of clarity during manuscript preparation. No AI tools were used for data analysis, interpretation of results, or generation of scientific content. All scientific conclusions, data accuracy, and final wording were reviewed and approved by the authors, who take full responsibility for the content of this manuscript.

## Disclosures

The authors have no conflicts of interest to disclose.
