# Supplementary material for: Combined impact of paced QRS duration and right ventricular pacing burden on heart failure admission: Identifying clinical progression to stage C/D in pacemaker recipients
Source: Heart Rhythm O2. 2026 Jul 21;7(10):1942–9. doi: 10.1016/j.hroo.2026.07.013 (PMC13700360; doi:10.1016/j.hroo.2026.07.013)
Supplement: Supplementary file 1 [file mmc1.docx]

Supplemental Table 1. Echocardiographic Changes During Follow-up

| Variable | Baseline | Follow-up | ∆(Follow-up-Baseline) | n | p-value |
| --- | --- | --- | --- | --- | --- |
| LVEF (%) | 60±13 | 56±13 | -5.0±11 | 200 | <0.001 |
| LVEDD (mm) | 48.3±8.2 | 47.2±9.1 | -1.1±6.4 | 197 | 0.013 |
| LVESD (mm) | 33.0±9.6 | 32.9±9.6 | 0.1±6.3 | 180 | 0.827 |
| RVd (mm) | 39±3 | 43±6 | - | 6/12 | -* |

Values are mean ± SD or n. Follow-up LVEF represents the most recent echocardiographic assessment during follow-up.

-* RV dimension was not routinely measured in our clinical practice and was available only in a small subset of patients (n=6-12). Therefore, RV size was not included in the statistical analysis.
